# Evaluating the Subjective Orgasm Experience Through Sexual Context, Gender, and Sexual Orientation

**DOI:** 10.1007/s10508-022-02493-3

**Published:** 2022-12-12

**Authors:** Laura Elvira Muñoz-García, Carmen Gómez-Berrocal, Juan Carlos Sierra

**Affiliations:** Mind, Brain, and Behaviour Research Center (CIMCYC), University of Granada, Campus Universitario de Cartuja s/n, 18011 Granada, Spain

**Keywords:** Subjective orgasm experience, Solitary masturbation, Sexual relationships, Gender, Sexual orientation

## Abstract

The subjective orgasm experience (SOE) is the psychological perception of orgasm sensations and closely related to sexual health. Here, SOE was studied through the context in which it is experienced (sexual relationships and solitary masturbation), gender, and sexual orientation. For this purpose, data were collected from 4255 people (1927 men and 2328 women) of different sexual orientations (heterosexual = 1545; bisexual = 1202; and gay = 1508) who completed two versions of the Orgasm Rating Scale (ORS) for both contexts (i.e., sexual relationships and solitary masturbation) along with a socio-demographic questionnaire. Results showed that the ORS in the context of solitary masturbation is an instrument invariant by gender and sexual orientation. Significant differences in SOE were found by context: it was more intense in the context of sexual relationships (vs. solitary masturbation); by gender: women (vs. men) reported greater intensity; and by sexual orientation, with heterosexual people (vs. gay and bisexual people) having a more intense experience.

## Introduction

Orgasm is described as a sensation of intense pleasure that produces an alteration of consciousness combined with changes in pelvic musculature and resolution of sexual vasocongestion, usually accompanied by a feeling of well-being and satisfaction (Meston et al., 2004). This feeling derives from physical or mental stimulation during the sexual activity and includes psychological, physiological, and social aspects (Levin & van Berlo, 2004). It is a normative indicator of sexual pleasure, as well as a healthy sexuality, and it is also associated with greater sexual satisfaction (Kontula & Miettinen, 2016; Leavitt et al., 2021). Here, the subjective orgasm experience (SOE) refers to the subjective assessment of orgasm sensations (Arcos-Romero & Sierra, 2018).

Studies on orgasm focused primarily on physiological responses, whereas SOE is less considered (Arcos-Romero & Sierra, 2018; Mah & Binik, 2005). Studying the SOE is necessary for understanding sexual satisfaction (Lawrance & Byers, 1995). Therefore, and given that sexual satisfaction is affected, among others, by individual and relational characteristics (Calvillo et al., 2018; Sánchez-Fuentes et al., 2014), it seems logical to analyze the SOE through personal (e.g., gender and sexual orientation) and contextual (e.g., context in which orgasm is experienced: solitary masturbation vs. sexual relationships) characteristics.

Instruments to assess the ease/difficulty of orgasm are common, although those that assess SOE are scarce (Arcos-Romero & Sierra, 2018). The Orgasm Rating Scale (ORS) developed by Mah and Binik (2002) and adapted to Spanish population by Arcos-Romero et al. (2018) is one exception. The original scale included two dimensions: Cognitive-affective, referring to the evaluative and affective experiences associated with orgasm, and Sensory, referring to the physiological sensations of orgasm (Mah & Binik, 2002). Based on this SOE model (Mah & Binik, 2002) Arcos-Romero et al. (2019) recently proposed a four-dimensional scale that assesses SOE in the context of sexual relationships. The four dimensions were Affective (emotions experienced during orgasm), Sensory (perception of physiological changes experienced during orgasm), Intimacy (intimate aspect of the experience), and Rewards (reinforcing effect of the orgasm). This four-dimension structure has been shown to be consistent as found in various psychometric studies (Arcos-Romero & Sierra, 2019; Cervilla et al., 2022; Mangas et al., 2022) and a laboratory study (Arcos-Romero et al., 2019) in both contexts (sexual relationships and solitary masturbation).

To date, most studies on SOE have focused on the context of sexual relationships (Arcos-Romero & Sierra, 2019, 2020; Arcos-Romero et al., 2018, 2019; Mah & Binik, 2001; Mangas et al., 2022). The SOE has hardly been studied in the context of solitary masturbation, probably because masturbation has been considered taboo for centuries (Das, 2007; Sierra et al., 2022). Furthermore, it is assumed that solitary sexuality is less complex, less context-dependent, and less desirable than partnered sexuality (Goldey et al., 2016).

The study of SOE in the context of solitary masturbation is of interest considering that this behavior can be a means to achieve sexual health (Coleman, 2003), and is important for a healthy sexual development (Bancroft et al., 2003; Das, 2007; Langfeldt, 1981). Although evidence shows an inverse relationship between masturbation and sexual relationships, supporting a compensatory purpose for solitary masturbation (Dekker & Schmidt, 2003; Kontula & Haavio-Mannila, 2002), there is also proof that solitary masturbation and sexual relationships are complementary and mutually reinforcing behaviors (Pinkerton et al., 2003). There is evidence that the SOE is perceived as more intense in the context of sexual relationships than in that of solitary masturbation (Bensman, 2011; Levin, 2007; Mah & Binik, 2002; Pinkerton et al., 2003; Santtila et al., 2007; Sierra et al., 2021). It has been shown that orgasmic satisfaction and orgasmic facility during sexual relationships are directly associated with positive attitude toward and the frequency of solitary masturbation (Cervilla et al., 2022; Palmer, 2014; Sierra et al., 2022). Therefore, we propose that the experience of masturbation, practiced in both contexts in a complementary manner, may be beneficial. In this sense, it is relevant to study the SOE when masturbation is practiced in different contexts (i.e., sexual relationships and solitary masturbation). Recent psychometric studies indicate that the ORS has the same structure—consisting of four dimensions (i.e., Affective, Sensory, Intimacy, and Rewards)—both in the contexts of sexual relationships (Arcos-Romero & Sierra, 2019) and solitary masturbation (Cervilla et al., 2022).

The experience associated with masturbation may depend not only on the context in which it is practiced, but also sexual gender norms may be important in this regard. Said norms prescribe and guide the differences between men and women in specific domains of sexuality (Gagnon & Simon, 2009; Kiefer & Sanchez, 2007). Considering that people internalize and enact these norms as part of the social identity they construct by identifying with a group (Hogg et al., 2004), we propose that both behavior and the experience of sexuality may be affected by a person's group affiliation. This group belonging may be defined by gender and sexual orientation. For example, there are differences between heterosexual men and women, or between non-heterosexual people relative to heterosexual people in how they understand sex, sexuality, and sexual health (Horowitz & Spicer, 2013; Rubinsky & Cooke-Jackson, 2018).

More precisely, gender can affect the experience of orgasm (Paterson et al., 2014) and the SOE (Arcos-Romero & Sierra, 2020; Mangas et al., 2022). Women reported greater intensity of SOE in both contexts of sexual relationships (Arcos-Romero & Sierra, 2018; Arcos-Romero et al., 2018; Mangas et al., 2022) and solitary masturbation (Sierra et al., 2021, 2022). The study on differences across gender suggested that orgasm is more complex in women (Colson, 2010). In this vein, in women, more than in men, the SOE is associated with a greater number of variables (Arcos-Romero & Sierra, 2020). Based on such findings, we proposed to study the differences between men and women in the dimensions that compose the SOE (i.e., Affective, Sensory, Intimacy and Rewards) both in the context of solitary masturbation and in the context of sexual relationships in romantic relationships. Previous studies adopting this approach demonstrated that in the context of sexual relationships, women scored higher than men in Affective and Sensory dimensions (Arcos-Romero et al., 2018, 2019), while men scored higher in the Rewards dimension (Arcos-Romero & Sierra, 2019). On the other hand, in the context of solitary masturbation, women had significantly higher scores on Intimacy dimension (Mah & Binik, 2002).

As mentioned above, the experience of sexuality can be affected by the internalization of gender norms that derive from the sexual orientation the person identifies with. This reality requires a more inclusive methodological approach that considers minority sexual orientations and not only heteronormative ones (Andersen & Zou, 2015; Blair, 2016). However, most studies in this field have been conducted with heterosexual people and in the context of sexual relationships (Arcos-Romero et al., 2019). While it is true that same-gender and mixed-gender couples’ sexual activities share a few similarities (Holmberg & Blair, 2009), it is a reality that the literature has adopted a heteronormative approach to study sexuality and it would be mistaken to presume that sexuality in same-gender partnerships adheres to these heteronormative frameworks (Blair et al., 2017; Scott et al., 2018). There are few studies that compare the orgasmic experience in people of different sexual orientations, but the results indicate that the person’s sexual orientation influences the orgasmic experience (Frederick et al., 2018; Garcia et al., 2014; Herbenick et al., 2010; Mangas et al., 2022). This evidence shows lesbian women experience more coupled orgasms than heterosexual and bisexual women do (Frederick et al., 2018, 2021; Garcia et al., 2014; Willis et al., 2018), while in men there’s no difference between sexual orientations (Frederick et al., 2018, 2021; Garcia et al., 2014).

Regarding the SOE, Mangas et al. (2022) recently studied for the first time the SOE in the context of sexual relationships in sexual minority people (gay men and lesbians). They found that heterosexual men scored higher than gay men in the Rewards dimension. This was explained by that, in contrast to gay men, who place more emphasis on the course or process of the sexual connection rather than the outcome of the same, heterosexual men place a greater value on the results or consequences of the orgasm achieved. Conversely, lesbians scored higher than heterosexual women in the Intimacy dimension. This was explained by the fact that lesbians typically exhibit great communication about their sexual life, which enhances intimacy and improves dyadic adjustment (Calvillo et al., 2020a, 2020b; Jordan & Deluty, 2000). This present study aimed to go further and analyze the SOE in both contexts (solitary masturbation and sexual relationships) including bisexual men and women, besides heterosexual and gay men and women.

To measure and compare a construct between different groups, it is essential to use invariant instruments (Muñiz et al., 2013; Muñiz & Fonseca-Pedrero, 2019). Factorial invariance establishes the precision with which an instrument measures the same construct among different groups (Pineda et al., 2018), which shows the construct has the same meaning for those groups or over several measurement circumstances (Putnick & Bornstein, 2016). Using this approach, this study analyzed the measurement invariance of the ORS in the context of solitary masturbation by gender and sexual orientation.

Therefore, the objectives of this study were, in the first place, to examine the measurement invariance by gender and sexual orientation of the Spanish version of the ORS (ORS; Mah & Binik, 2011) of Cervilla et al. (2022), which assesses SOE in the context of solitary masturbation. Based on previous data (Mangas et al., 2022), where this structure in the context of sexual relationships was invariant by gender and sexual orientation, we hypothesized that it would be invariant by gender and sexual orientation (H1). Second, SOE was compared in the context of solitary masturbation and that of sexual relationships. We predicted SOE would be more intense in the context of sexual relationships than in the context of solitary masturbation (Goldey et al., 2016; Sierra et al., 2021) (H2). Third, we examined the relationship between gender and SOE in both contexts (solitary masturbation and sexual relationships). A significant relationship between gender and SOE was expected in both contexts (solitary masturbation and sexual relationships): women (vs. men) would present higher scores on SOE (Arcos-Romero & Sierra, 2019; Arcos-Romero et al., 2018; Sierra et al., 2021, 2022) (H3). Lastly, we examined the relationship between sexual orientation (heterosexual, bisexual, and gay) and SOE in both contexts (solitary masturbation and sexual relationships). A significant relationship between sexual orientation and SOE was expected in both contexts (solitary masturbation and sexual relationships): heterosexual people (vs. bisexual and gay people) would have the highest SOE scores (Mangas et al., 2022) (H4).

## Method

### Participants

This study was conducted in two separate samples. The first sample (Table 1) was used to test H1 and consisted of 2233 Spanish adults (1079 men and 1154 women) aged between 18 and 78 years (*M* = 35.09; *SD* = 12.56). Members were classified according to their Kinsey scale scores as heterosexual (scores = 1 and 2; *n* = 804), bisexual (scores = 3, 4, and 5; *n* = 669), and gay (scores = 6 and 7; *n* = 760). In terms of education level, 73.1% went to university, 23.8% reached secondary education, 3% reached primary education and 0.1% had no education. The second sample (Table 2) was used to test the rest of the hypotheses and included 2,022 Spanish adults (848 men and 1174 women) aged between 18 and 74 years (*M* = 28.64; *SD* = 9.34). According to the Kinsey scale, they were classified as heterosexual (scores = 1 and 2; *n* = 741), bisexual (scores = 3, 4, and 5; *n* = 533), and gay (scores = 6 and 7; *n* = 748). In terms of education level, 77.1% went to university, 21.2% reached secondary education, 1.5% reached primary education and 0.2% had no education. For both samples, the inclusion criteria were being aged more than 18 years, of Spanish nationality, cisgender, having masturbated, and having had a sexual relationship at some point.

**Table 1:** Sociodemographic characteristics by gender and sexual orientation from sample 1

	Heterosexual		Bisexual		Gay	
	Men *n* = 402	Women *n* = 402	Men *n* = 295	Women *n* = 374	Men *n* = 382	Women *n* = 378
Age *M *(SD)	41.47 (12.76)	40.05 (12.18)	29.05 (11.66)	28.17 (10.05)	38.40 (11.86)	31.22 (9.13)
*Sexual activity in the relationship in the last 3 months n (%)*						
Yes	293 (93.3)	264 (95.7)	117 (93.6)	215 (96)	188 (92.6)	226 (95.8)
No	21 (6.7)	12 (4.3)	8 (6.4)	9 (4)	15 (7.4)	10 (4.2)
*Sexual activity without a partner in the last 3 months n (%)*		6.11*				
Yes	50 (60.2)	91 (76.5)	98 (59.8)	81 (55.5)	135 (75.8)	72 (51.8)
No	33 (39.8)	28 (23.5)	66 (40.2)	65 (44.5)	43 (24.2)	67 (48.2)
*Current masturbation frequency n (%)*						
More than once a day	23 (5.7)	11 (2.7)	27 (9.2)	5 (1.3)	35 (9.2)	8 (2.1)
Once a day	76 (18.9)	23 (5.7)	68 (23.1)	36 (9.6)	98 (25.7)	28 (7.4)
A few times a week	47 (11.6)	176 (43.8)	180 (61)	225 (60.1)	200 (52.5)	206 (54.6)
A few times a month	226 (56.1)	135 (33.6)	14 (4.7)	79 (21.1)	36 (9.4)	97 (25.7)
Less than once a month	18 (4.5)	45 (11.2)	5 (1.7)	28 (7.5)	11 (2.9)	32 (8.5)
Never	12 (3)	12 (3)	1 (0.3)	1 (0.3)	1 (0.3)	6 (1.6)

**Table 2:** Sociodemographic characteristics by gender and sexual orientation from sample 2

	Heterosexual		Bisexual		Gays	
	Men *n* = 315	Women *n* = 426	Men *n* = 133	Women *n* = 400	Men *n* = 400	Women *n* = 348
Age *M (SD)*	30.94 (11.62)	26.12 (7.76)	25.38 (8.46)	24.63 (6.16)	34.01 (10.26)	29.32 (7.14)
*Sexual activity in the relationship in the last 3 months n (%)*						
Yes	245	318	78	283	225	272
No	–	–	–	–	–	–
*Sexual activity without a partner in the last 3 months n (%)*						
Yes	70	108	55	117	175	76
No	–	–	–	–	–	–
*Current masturbation frequency n (%)*						
More than once a day	15 (12.3)	7 (1.7)	11 (8.9)	8 (2)	36 (9.1)	7 (2)
Once a day	38 (31.1)	35 (8.3)	34 (27.6)	38 (9.5)	107 (27)	27 (7.8)
A few times a week	58 (47.5)	217 (51.1)	74 (60.2)	238 (59.6)	217 (54.6)	182 (52.5)
A few times a month	8 (6.5)	127 (29.9)	4 (3.3)	88 (22.1)	27 (6.8)	90 (25.9)
Less than once a month	2 (1.6)	30 (7.1)	–	23 (5.8)	9 (2.3)	36(10.4)
Never	1 (0.8)	8 (1.9)	–	4 (1)	1 (0.3)	5 (1.4)

### Procedure

Participants were evaluated online, a common procedure for assessing sexual behaviors (Calvillo et al., 2020a, Cervilla et al., 2021). The online survey was distributed using virtual platforms (Facebook, Twitter, WhatsApp, and e-mail). Previous studies confirmed no differences to the traditional paper-and-pencil method (Álvarez-Muelas et al., 2021; Sierra et al., 2018).

Participants signed an informed consent form containing the aim and purpose of the study. Anonymity, data protection, and confidentiality were guaranteed. Automatic responses were avoided by answering a simple random arithmetic question. The data were thoroughly reviewed to rule out any cases with inconclusive responses or abnormal patterns. The study was conducted according to the guidelines of the Declaration of Helsinki and approved by the University of Granada Human Research Ethics Committee [2594/CEIH/2022].

### Measures

Sociodemographic and Sexual History Questionnaire. This questionnaire collected information on gender, age, nationality, educational level (No studies, Primary education, Secondary education, University), partner relationships, age at first sexual relationship, solitary and partnered sexual activity (Yes, No), and current frequency of masturbation, ranging from Never to More than once a day. (Table 1)

The Kinsey scale (Kinsey et al., 1998) evaluated sexual orientation through seven items ranging from 1 (Exclusively heterosexual) to 7 (Exclusively homosexual).

The Spanish version of the ORS (Mah & Binik, 2011) by Cervilla et al. (2022) evaluated the SOE of the last orgasm they experienced in the context of solitary masturbation. It consists of 25 adjectives answered on a 6-point Likert-type scale ranging from 0 (Does not describe it at all) to 5 (Describes it perfectly). The items were grouped into four subscales: Affective (e.g., blissful), Sensory (e.g., throbbing), Intimacy (e.g., loving), and Rewards (e.g., soothing). Internal consistency reliability coefficients ranged from 0.71 (Intimacy) to 0.95 (Sensory). In this study, they were 0.85 for Affective, 0.94 for Sensory, 0.69 for Intimacy, and 0.81 for Rewards.

The Spanish version of the ORS (ORS; Mah & Binik, 2011) by Arcos-Romero et al. (2018) assessed the SOE of the last orgasm they experienced in the context of sexual relationships. It has the same items and subscales as the ORS for the context of solitary masturbation: Affective, Sensory, Intimacy, and Rewards. Internal consistency reliability coefficients ranged from 0.82 (Intimacy) to 0.95 (Sensory). In this study, they were 0.85 for Affective, 0.91 for Sensory, 0.82 for Intimacy, and 0.84 for Rewards. This version has been shown to be a measure invariant to gender and sexual orientation (Arcos-Romero & Sierra, 2019).

### Data Analysis

First, to impute missing data, a nonparametric imputation method was used. It built a random forest model for each variable, then used the model to predict missing values in each of these variables with the help of observed values. Then, the factorial invariance by gender and sexual orientation of the ORS in the context of solitary masturbation was examined using the factorial structure from Cervilla et al. (2022) as reference. The Weighted Least Squares Measurement (WLSM) estimation method with chi-square adjustment of the mean was adopted. In concordance with recent suggestions (Tarka, 2017), the WLSM estimation method is a robust estimator of non-compliance with multivariate normality for ordinal/categorical data (Gana & Broc, 2019). Root mean squared error of approximation (RMSEA) values less than 0.06, and comparative fit index (CFI) and Tucker–Lewis index (TLI) values greater than 0.90 indicate a good fit. Factor invariance was progressively analyzed at four levels: Configural, Weak, Strong, and Strict. Recommendations on CFI as the main invariance fit (Milfont & Fischer, 2010; Putnick & Bornstein, 2016) were followed to accept the equivalence of the models for the different levels. A change in CFI equal to or greater than 0.01 allows adopting the less constrained model and rejecting the more restrictive one.

Next, multivariate analyses of variance (MANCOVAs) were performed for each of the four dimensions of the ORS (Affective, Sensory, Intimacy, and Rewards) as dependent variables, considering the context as a within-subject factor (solitary masturbation vs. sexual relationships), and gender (men vs. women) and sexual orientation (heterosexual vs. bisexual vs. gay) as between-subject factors. Finally, to examine between-group differences, analyses of variance (ANOVAs) and Bonferroni post hoc tests were performed. In all cases, the requirements for a MANCOVA were evaluated. Age was controlled as a covariate.

The measurement invariance analyses were performed in the R® environment (version 3.6.3; R Core Team, 2020) with the RStudio® interface (version 1.2.5042; RStudio Team, 2020). The missForest (version 1.4; Stekhoven & Bühlmann, 2011) packages were used to impute missing data and lavaan for the measurement invariance (Rosseel, 2012). The remaining analyses were performed using IBM SPSS Statistics analytical software.

## Results

### Factorial Invariance by Gender and Sexual Orientation

The factorial structure of the ORS for the context of solitary masturbation presented strict invariance, both by gender (RMSEA = 0.055 [0.054, 0.057], CFI = 0.977) and sexual orientation (RMSEA = 0.053 [0.052, 0.055], CFI = 0.978), as shown in Table 3.

**Table 3:** Measurement of invariance across gender and sexual orientation of the ORS in the context of solitary masturbation

Model	*χ*^2^	*df*	*p*	CFI	TLI	RMSEA	RMSEA CI 90%	ΔCFI
*Gender (men, women)*								
Configural	5094.39	538	< .01	.980	.978	.054	[.053, .056]	
Weak	4274.17	559	< .01	.979	.977	.055	[.054, .057]	< .01
Strong	4502.83	580	< .01	.977	.977	.056	[054, .057]	< .01
Strict	4575.70	605	< .01	.977	.977	.055	[0.054, .057]	< .01
*Sexual orientation (heterosexual, bisexual, gay)*								
Configural	5413.72	807	< .01	.980	.978	.054	[.053, .056]	
Weak	4241.80	849	< .01	.979	.978	054	[.052, .056]	< .01
Strong	4401.97	891	< .01	.979	.979	.053	[.052, .055]	< .01
Strict	4581.62	941	< .01	.978	.979	.053	[.052, .055]	< .01

### Comparison Between Contexts

The results of the mixed MANCOVA revealed significant differences [*F*(1, 2015) = 45.13, *p* < 0.001; Pillai's Trace = 0.02] in the Affective dimension between the solitary masturbation (*M* = 23.92, *SD* = 5.07) and sexual relationships contexts (*M* = 26.47, *SD* = 4.18). The differences obtained in the Sensory dimension [*F*(1, 2015) = 56.75, *p* < 0.001; Pillai's Trace = 0.03] indicated higher scores in the sexual relationships (*M* = 41.12, *SD* = 13.72) than solitary masturbation context (*M* = 32.34, *SD* = 16.49). In the Intimacy dimension, significant differences were also obtained [*F*(1, 2015) = 177.46, *p* < 0.001; Pillai's Trace = 0.08], with higher scores in the sexual relationships (*M* = 10.24, *SD * = 3.89) than solitary masturbation context (*M* = 6.16, *SD* = 3.53). Finally, in the Rewards dimension [*F*(1, 2015) = 13.15, *p* < 0.001; Pillai's Trace = 0.006], the score was higher in the solitary masturbation (*M* = 11.08, *SD* = 3.54) than sexual relationships context (*M* = 10.13, *SD* = 3.98). Age was not a significant multivariate covariate in Affective [*F*(1, 2015) = 0.16, *p* = 0.69; Pillai's Trace < 0.001], Sensory [*F*(1, 2015) = 0.35, *p* = 0.56; Pillai's Trace < 0.001], Intimacy [*F*(1, 2015) = 0.92, *p* = 0.34; Pillai's Trace < 0.001] or Rewards [*F*(1, 2015) = 0.23, *p* = 0.63; Pillai's Trace < 0.001].

### Comparison by Gender

**Fig. 1:**
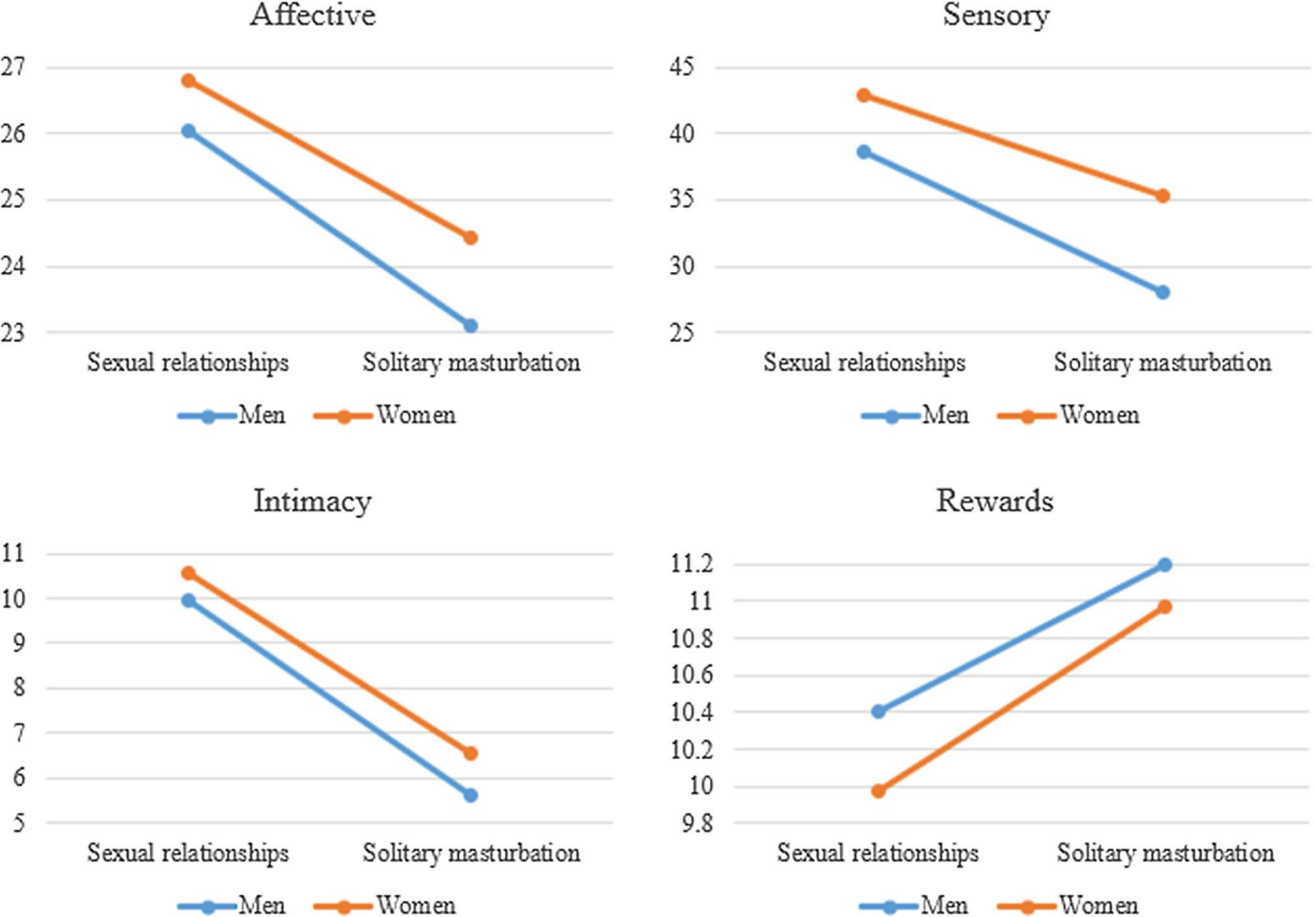
Mean scores across gender and context for each dimension of subjective orgasmic experience. Values represented correspond to marginal means

The mixed MANCOVA within-subject contrast test showed a significant effect on the Affective dimension [*F*(1, 2015) = 4.76, *p* = 0.03; Pillai's Trace = 0.002], so the mean score in this dimension varied between the two contexts for both men and women. The univariate test results for men [(*F*(1, 2015) = 196.57, *p* < 0.001, *d* = 0.60)] and women [(*F*(1, 2015) = 211.90, *p* < 0.001, *d* = 0.52)] indicated that for both, the score was significantly higher in the sexual relationships (men: *M* = 26.02, *SD* = 4.37; women: *M* = 26.79, *SD* = 4.00) than solitary masturbation context (men: *M* = 23.15, *SD* = 5.18; women: *M* = 24.49, *SD* = 4.91) (see Fig. 1). The MANCOVA test for between-subject effects pointed to significant differences between men and women in this dimension [*F*(1, 2015) = 32.62, *p* < 0.001, *ηp*^*2*^ = 0.02]. Specifically, the univariate ANCOVAs showed significant differences between men (*M* = 23.15, *SD* = 5.18) and women (*M* = 24.49, *SD* = 4.91) in the solitary masturbation context: *F*(1, 2015) = 29.35, *p* < 0.001, *d* = 0.27. In the sexual relationships context, differences between men (*M* = 26.02, *SD* = 4.37) and women (*M* = 26.80, *SD* = 4.00) were also significant: *F*(1, 2015) = 13.62, *p* < 0.001, *d* = 0.19. In both contexts, scores are higher for women (vs. men).

In the Sensory dimension, the mixed MANCOVA within-subject contrast test indicated a significant effect [*F*(1, 2015) = 17.45, *p* < 0.001; Pillai's Trace = 0.009], such that the mean score varied between the two contexts for both men and women. The univariate tests were significant for men [(*F*(1, 2015) = 353.38, *p* < 0.001, *d* = 0.73] and women [(*F*(1, 2015) = 304.55, *p* < 0.001, *d* = 0.49]. For both genders, the scores were significantly higher in the sexual relationships context (men: *M* = 38.77, *SD* = 13.94; women: *M* = 42.82, *SD* = 13.30) than solitary masturbation context (men: *M* = 28.01, *SD* = 15.39; women: *M* = 35.46, *SD* = 16.57) (see Fig. 1). The MANCOVA between-subject effects test showed significant differences between men and women on this dimension [*F*(1, 2015) = 73.74, *p* < 0.001, *ηp*^*2*^ = 0.04]. Specifically, the univariate ANCOVAs indicated significant differences between men (*M* = 28.01, *SD* = 15.39) and women (*M* = 35.46, *SD* = 16.57) in the solitary masturbation context (*F*(1, 2015) = 77.83, *p* < 0.001, *d* = 0.47), and sexual relationships context (*F*(1, 2015) = 34.99, *p* < 0.001, *d* = 0.30; men (*M* = 38.77, *SD* = 13.94), women (*M* = 42.82, *SD* = 13.30)). The scores were higher for women.

In the Intimacy dimension, the mixed MANCOVA within-subjects contrast test showed no significant effect: *F*(1, 2015) = 2.73, *p* = 0.09; Pillai's Trace = 0.001. However, the univariate test results for men [*F*(1, 2015) = 663.96, *p* < 0.001, *d* = 1.16] and women [*F*(1, 2015) = 938.87, *p* < 0.001, *d* = 1.07] indicated significantly higher scores in the sexual relationships context (men: *M* = 9.87, *SD* = 4.01; women: *M* = 10.51, *SD* = 3.78) than in the solitary masturbation context (men: *M* = 5.63, *SD* = 3.30; women: *M* = 6.55, *SD* = 3.64) (see Fig. 1). The MANCOVA test for between-subject effects indicated significant differences between men and women on this dimension: *F*(1, 2015) = 38.37, *p* < 0.001, *ηp*^2^ = 0.02. Specifically, the univariate ANCOVAs showed significant differences between men (*M* = 5.63, *SD* = 3.30) and women (*M* = 6.55, *SD* = 3.64) in the solitary masturbation context (*F*(1, 2015) = 39.11, *p* < 0.001, *d* = 0.26) and sexual relationships context between men (*M* = 9.87, *SD* = 4.00) and women (*M* = 10.51, *SD* = 3.78): *F*(1, 2015) = 14.38, *p* = 0.001, *d* = 0.16. Scores were higher for women (vs. men).

Finally, in the Rewards dimension, the mixed MANCOVA within-subject contrast test showed no significant effect: *F*(1, 2015) = 1.17, *p* = 0.28; Pillai's Trace = 0.001. However, the results of the univariate tests comparing the two contexts for men [*F*(1, 2015) = 31.56, *p* < 0.001, *d* = 0.24] and women [*F*(1, 2015) = 82.09, *p* < 0.001, *d* = 0.30] indicated significantly higher scores in the solitary masturbation context (men: *M* = 11.21, *SD* = 3.35; women: *M* = 10.98, *SD* = 3.66) than sexual relationships context (men: *M* = 10.36, *SD* = 3.85; women: *M* = 9.97, *SD* = 4.06) (Fig. 1). The MANCOVA test for between-subject effects indicated significant differences between men and women on this dimension: *F*(1, 2015) = 4.12, *p* = 0.04, *ηp*^2^ = 0.002. Specifically, the univariate ANCOVAs showed significant differences between men (*M* = 10.36, *SD* = 3.85) and women (*M* = 9.97, *SD* = 3.06) only in the sexual relationships context: *F*(1, 2015) = 4.78, *p* = 0.03, *d* = 0.11. The scores for men were higher (vs. women).

### Comparison by Sexual Orientation

**Fig. 2:**
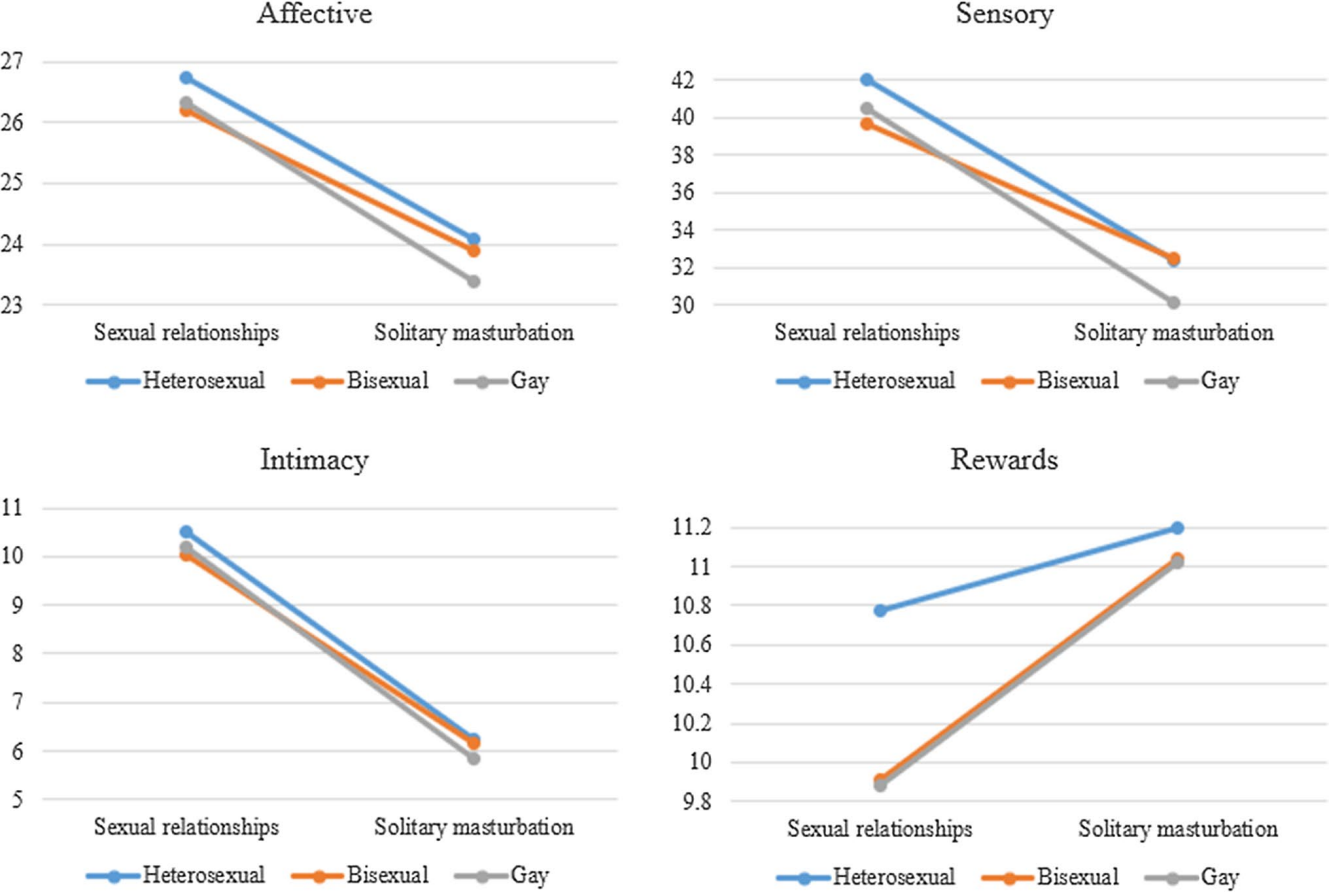
Mean scores across gender and context for each dimension of subjective orgasmic experience. Values represented correspond to marginal means

In the Affective dimension, the mixed MANCOVA within-subject contrast test indicated no significant effect [*F*(2, 2015) = 1.91, *p* = 0.15; Pillai's Trace = 0.002], suggesting that the mean score did not vary between the two contexts in each group classified by sexual orientation. However, the results of the univariate tests comparing the two contexts for each sexual orientation showed that in all three groups—heterosexual [*F*(1, 2015) = 173.82, *p* < 0.001, *d* = 0.56], bisexual [*F*(1, 2015) = 68.59, *p* < 0.001, *d* = 0.42], and gay [*F*(1, 2015) = 212.75, *p* < 0.001, *d* = 0.63]—the mean in the context of sexual relationships [heterosexual (*M* = 26.76, *SD* = 3.78), bisexual (*M* = 26.32, *SD* = 4.29), and gay (*M* = 26.28, *SD* = 4.44)] was significantly higher than in the solitary masturbation context [heterosexual (*M* = 24.20, *SD* = 5.22), bisexual (*M* = 24.33, *SD* = 5.13), and gay (*M* = 23.36, *SD* = 4.82)] (Fig. 2). The MANCOVA test for between-subject effects confirmed the significant effect of sexual orientation: *F*(2, 2015) = 4.17, *p* = 0.016, *ηp*^2^ = 0.004. Specifically, the univariate ANCOVAs showed significant differences between heterosexual, bisexual, and gay people in the solitary masturbation context: scores were higher for heterosexual and bisexual people (vs. gay people). No differences were found between the three groups in the context of sexual relationships (Table 4).

**Table 4:** Results of tests of effects between subjects of the multivariate analysis of covariance

	Heterosexual	Bisexual	Gay			
	*M* (*SD*)	*M* (*SD*)	*M* (*SD*)	*F* (*df*)	*ηp*^2^	Post hoc contrasts between subjects
*Affective*						
Sexual relationships	26.77 (3.78)	26.32 (4.30)	26.28 (4.44)	2.91 (2, 2015)	.003	*n.s*
Solitary masturbation	24.20 (5.22)	24.33 (5.13)	23.36 (4.82)	3.72* (2, 2015)	.004	Heterosexual and bisexual > gay*
*Sensory*						
Sexual relationships	42.23 (13.74)	40.72 (13.41)	40.32 (13.86)	5.08* (2, 2015)	.005	Heterosexual and bisexual > gay*
Solitary masturbation	33.06 (17.08)	34.70 (16.39)	29.93 (15.67)	3.11** (2, 2015)	.003	Heterosexual and bisexual > gay*
*Intimacy*						
Sexual relationships	10.47 (3.71)	10.12 (3.89)	10.10 (4.06)	2.48 (2, 2015)	.002	*n.s*
Solitary masturbation	6.27 (3.48)	6.49 (3.59)	5.82 (3.51)	5.09 (2, 2015)	.005	*n.s*
*Rewards*						
Sexual relationships	10.70 (3.78)	9.72 (4.16)	9.86 (3.98)	11.28*** (2, 2015)	.01	Heterosexual > bisexual** and gay***
Solitary masturbation	11.18 (3.57)	11.01 (3.73)	11.02 (3.36)	.56 (2, 2015)	.001	*n.s*

*p < .05; **p < .01; ***p < .001

In the Sensory dimension, the mixed MANCOVA within-subject contrast test presented a significant effect: *F*(2, 2015) = 5.57, *p* = 0.004; Pillai's Trace = 0.006. The univariate test results for the three groups—heterosexual [*F*(1, 2015) = 313.54, *p* < 0.001, *d* = 0.59], bisexual [*F*(1, 2015) = 95.07, *p* < 0.001, *d* = 0.40], and gay [*F*(1, 2015) = 357.54, *p* < 0.001, *d* = 0.70]—showed that the mean in the context of sexual relationships [heterosexual (*M* = 42.23, *SD* = 13.74), bisexual (*M* = 40.72, *SD* = 13.41), and gay (*M* = 40.32, *SD* = 13.86)] was significantly higher than in the solitary masturbation context [heterosexual (*M* = 33.06, *SD* = 17.08), bisexual (*M* = 34.70, *SD* = 16.39), and gay (*M* = 29.93, *SD* = 15.67] (Fig. 2). The MANCOVA test for between-subject effects confirmed the significant effect of sexual orientation: *F*(2, 2015) = 3.39, *p* = 0.03, *ηp*^2^ = 0.003. Specifically, the univariate ANCOVAs demonstrated significant differences between heterosexual, bisexual, and gay people in both contexts, with heterosexual and bisexual people scoring higher than gay people (Table 4).

In the Intimacy dimension, the mixed MANCOVA within-subject contrast test was not significant: *F*(2, 2015) = 1.62, *p* = 0.19; Pillai's Trace = 0.002. However, the univariate test results for each sexual orientation showed the significant effect of this context on all three groups: heterosexual [*F*(1, 2015) = 689.24, *p* < 0.001, *d* = 1.17], bisexual [*F*(1, 2015) = 300.49, *p* < 0.001, *d* = 0.97], and gay [*F*(1, 2015) = 705.07, *p* < 0.001, *d* = 1.13]. The mean was higher in the sexual relationships context [heterosexual (*M* = 10.47, *SD* = 3.71), bisexual (*M* = 10.12, *SD* = 3.88), and gay (*M* = 10.10, *SD* = 4.06)] than in the masturbation context [heterosexual (*M* = 6.27, *SD* = 3.48), bisexual (*M* = 6.49, *SD* = 3.59), and gay (*M* = 5.82, *SD* = 3.51) (Fig. 2). Although the MANCOVA test for between-subject effects indicated the significant effect of sexual orientation [*F*(2, 2015) = 4.80, *p* = 0.008, *ηp*^2^ = 0.005], the univariate ANCOVAs showed no significant differences between heterosexual, bisexual, and gay people in either context (Table 4).

Finally, in the Rewards dimension, the mixed MANCOVA within-subject contrast test yielded a significant effect [*F*(2, 2015) = 8.20, *p* < 0.001; Pillai's Trace = 0.008]. The results of the univariate tests comparing the two contexts for each sexual orientation showed the significant effect of the sexual relationships context: heterosexual [*F*(1, 2015) = 9.66, *p* = 0.002, *d* = 0.13], bisexual [*F*(1, 2015) = 34.94, *p* < 0.001, *d* = 0.32], and gay [*F*(1, 2015) = 69.21, *p* < 0.001, *d* = 0.31]. In all three groups, the mean was higher for the solitary masturbation context [heterosexual (*M* = 11.18, *SD* = 3.57), bisexual (*M* = 11.00, *SD* = 3.73), and gay (*M* = 11.02, *SD* = 3.36)] than the sexual relationships context [heterosexual (*M* = 10.69, *SD* = 3.78), bisexual (*M* = 9.72, *SD* = 4.16), and gay (*M* = 9.86, *SD* = 3.98)] (Fig. 2). In the between-subject effects test, the MANCOVA showed no significant effect of sexual orientation: *F*(2, 2015) = 5.95, *p* = 0.003, *ηp*^2^ = 0.006. However, the univariate ANCOVAs indicated significant differences between heterosexual, bisexual, and gay people in the sexual relationships context, such that heterosexual people (vs. bisexual and gay people) scored higher (Table 4).

## Discussion

The main objective of this study was first, to test measurement invariance by gender and sexual orientation of the Spanish version of the ORS (Mah & Binik, 2011) of Cervilla et al. (2022) in the context of solitary masturbation. Second, it was to analyze the SOE across situational (i.e., context in which orgasm was experienced: solitary masturbation vs. sexual relationships) and individual characteristics (i.e., gender and sexual orientation).

The results of the measurement invariance by gender and sexual orientation of the ORS in the solitary masturbation context confirmed that it is an invariant scale (H1), both by gender and sexual orientation. Therefore, it is a valid instrument to measure and compare the SOE of different groups (men vs. women, and heterosexual vs. bisexual vs. gay) (Pineda et al., 2018).

Previous studies indicated that the SOE was more intense in the context of sexual relationships (vs. that of solitary masturbation) (Bensman, 2011; Levin, 2007; Mah & Binik, 2002; Pinkerton et al., 2003; Santtila et al., 2007; Sierra et al., 2022). Although our results in general confirmed this pattern (H2), they also allowed us to qualify the role of context on the SOE. The results showed that the dimensions related to emotions, sensations, and intimacy were more intense in sexual relationships, while that related to the rewarding effect of orgasm was more intense in solitary masturbation. Specifically, the scores for the Affective, Sensory, and Intimacy dimensions were higher in the sexual relationships context (vs. solitary masturbation), while the scores for the Rewards dimension were more intense in the solitary masturbation context (vs. sexual relationships). Moreover, these results were repeated when the variables of an individual’s gender and sexual orientation are considered. This pattern confirmed the results of previous studies, which indicated that both men and women value partnered orgasms as more intimate and solitary orgasms as more rewarding (Mah & Binik, 2002).

Based on previous research, we expected to find a significant relationship between gender and the SOE dimensions in both contexts (Arcos-Romero & Sierra, 2019; Arcos-Romero et al., 2018; Sierra et al., 2021, 2022). In accordance with these studies (H3), women got higher scores than men on three dimensions (Affective, Sensory, and Intimacy) in the sexual relationships context, which refers to emotions, physiological changes, and the intimate aspect of the orgasmic experience. This is consistent with previous evidence showing that women associated orgasms achieved through sexual relationships with more intense bodily sensations, more intimacy, and greater connection in sexual relationships (Fahs, 2014). However, we found that on the Rewards dimension, in the context of sexual relationships, men scored higher than women, which is also consistent with previous studies in which men reported having a more rewarding orgasm (Paterson et al., 2014). Finally, no differences were found between men and women on the Rewards dimension in the solitary masturbation context. Thus, in this context, gender did not influence the rewarding aspect of orgasm.

One explanation for the gender gap in orgasm could be the idea that traditional heteronormative sexual scripts seem to grant men more agency than women, encouraging sexual acts that are more likely to produce orgasms in men (such as penile–vaginal intercourse) (Blair et al., 2017). In addition, the fact that the dimensions where women score higher than men are the ones related to emotions and intimacy is consistent with traditional sexual scripts where women are typically depicted as sexual gatekeepers who prioritize emotional closeness and fidelity. On the other hand, men scored higher in the rewarding dimension, also congruent with traditional sexual scripts where they seek a more physical aspect of sex (Masters et al., 2013). Herein, a dichotomous, antagonistic paradigm of heterosexuality is produced by the confluence of the opposing discourses in which men are pursuing subjects, while women are passive objects (Tolman, 2006). According to this perspective, female sexuality does not exist unless it results from emotional closeness and commitment to a relationship (Masters et al., 2013). Also, women may have higher evaluations when tested in research settings because they may have lower aspirations for sexual satisfaction (McClelland, 2010).

Traditionally, studies on SOE have been conducted on heterosexual people and in the context of sexual relationships (Arcos-Romero & Sierra, 2018, 2019, 2020; Arcos-Romero et al., 2018, 2019; Mah & Binik, 2001). To analyze the SOE in non-majority sexual orientations, this study included bisexual and gay people, expecting that as postulated in H4, heterosexual people would present higher scores (Frederick et al., 2018; Garcia et al., 2014). Our results showed that heterosexual, bisexual, and gay people differ on two dimensions of the solitary masturbation context (Affective and Sensory) and two dimensions in the sexual relationships context (Sensory and Rewards), partially confirming H4. The intensity of the SOE was always higher for heterosexual people than gay people, which clearly shows the need to consider sexual orientation when conducting studies on SOE, and that there were more differences between heterosexual people and gay people than between heterosexual and bisexual people. The fact that gay people reported lower SOE intensity than heterosexual and bisexual people specifically in the dimensions more related to physical experiences could be because people with same-sex partners tend to place less emphasis on the consequences, instead concentrating on the process or development of the sexual relationship rather than its outcome (Mangas et al., 2022). This may be supported by findings that suggest that same-sex couples exhibit higher levels of emotional closeness than heterosexual couples (Spitalnick & McNair, 2005), which may cause them to place a higher priority on the emotional aspects of a relationship (Mangas et al., 2022). Research also shows that queer women prioritize non-genital sexual acts like kissing, snuggling, and hugging even though orgasm is less likely to occur because of them alone (Garnets & Peplau, 2006) and even do not mention orgasm at all when describing their best sexual encounters (Chatterji et al., 2017).

Many studies conducted among gay population focus on a binary conception of sexual orientation, in which same-sex and other-sex attraction are presented as the only categories (Bradford, 2004). Because of this, people who identify as bisexual experience a unique form of stigmatization and discrimination called “biphobia” (Bradford, 2004), which stems from both the heteronormative society and LGBTIQ+ community, thus experiencing double discrimination (Brewster & Moradi, 2010; Mitchell et al., 2014). Currently, there is no information regarding the SOE of this group. In our study, we observed that bisexual people present similar scores to heterosexual people, surpassing scores of gay people in the Affective and Sensory dimensions of the solitary masturbation context, and in the Sensory dimension of the sexual relationships context. However, in the Rewards dimension of orgasm, in the sexual relationships context, they presented lower scores than heterosexual people. Considering that bisexuality is a minority orientation and is more invisible than a gay orientation, this finding could be related to the minority stress theory (Meyer, 1995), in which internalized homophobia is included as one of the processes that compose it (Meyer, 2003). However, it is not currently possible to confirm this relation because of the scarcity of data on aspects of bisexual people’s sexuality. Finally, in the remaining dimensions (Affective in the sexual relationships context, Intimacy in both contexts, and Rewards in the solitary masturbation context), no significant differences were found according to sexual orientation. Thus, we conclude that the differences by orientation in SOE are not generalized but dependent on the context and dimension studied.

### Limitations and Future Research Directions

One limitation of this study was that the sample was collected using a convenience non-probability sampling technique in an online format. In addition, for bisexual people when answering the ORS, the gender of the sexual partner with whom they had the orgasm they were rating was not asked, which would have been an interesting addition. Another limitation is that no information was asked about how orgasm was obtained, which would also have added an interesting nuance. Finally, the use of the Kinsey scale to measure sexual orientation is a limitation since it reduces sexual orientation to a purely behavior matter. In this regard, bisexuality was not considered as all the responses correspondent with plurisexual orientations, but only a subset limited to the responses 3 (Predominantly heterosexual, but more than incidentally homosexual), 4 (Bisexual), and 5 (Predominantly homosexual, but more than incidentally heterosexual).

Future research should consider the influence of gender roles and attitudes toward sexual gender norms to understand and explain the processes underlying the differences between men and women in the SOE and across contexts. It should also examine and identify the factors that may be causing lower scores of SOE of people with a minority sexual orientation, which will allow the implementation of more effective programs to promote the sexual health of individuals regardless of sexual orientation (Garcia et al., 2014). Likewise, it is necessary to keep in mind that addressing dysfunctions or problems related to any aspect of orgasm should be framed considering an approach focusing on both the gender and sexual orientation of the person.

## Conclusions

This study innovated analyzing the differences by context, gender, and sexual orientation of SOE with a focus on each of its dimensions (Affective, Sensory, Intimacy, and Rewards), having previously demonstrated the measurement invariance of the scale that measures it. Previous findings have confirmed differences between contexts and gender. Regarding context, although the SOE was generally more intense in the sexual relationships context, this depended on the dimension in which it was observed, indicating that future studies should continue to include the context of solitary masturbation. Regarding gender—for which more differences were found—women generally experienced more intensity. In addition, the inclusion of participants belonging to the LGBTIQ+ community showed that heterosexual people experienced the greatest SOE intensity in specific dimensions of both contexts.

## Data Availability

The data presented in this study are available on request from the corresponding author, J.C.S.
